# Muscle ultrasound to diagnose sarcopenia in chronic kidney disease: a systematic review and bayesian bivariate meta-analysis

**DOI:** 10.1186/s12882-023-03445-2

**Published:** 2024-01-04

**Authors:** Qinbo Yang, Chen Zhang, Zhuyun Zhang, Baihai Su

**Affiliations:** 1 Department of Nephrology, West China Hospital, Sichuan University, 610041 Chengdu, China; 2 West China Hospital, West China School of Medicine, Sichuan University, 610041 Chengdu, China; 3 Med+ Biomaterial Institute of West China Hospital/West China School of Medicine, Sichuan University, Guoxue Alley No. 37, Chengdu, Sichuan Province, 610041 Chengdu, China

**Keywords:** Sarcopenia, Ultrasonography, CKD, Chronic kidney disease, meta-analysis

## Abstract

**Objective:**

The aim of this systematic review was to assess the diagnostic test accuracy of muscle ultrasound for sarcopenia among chronic kidney disease (CKD) populations.

**Background:**

Sarcopenia has become a worldwide health issue, especially for CKD patients. Conventional techniques of muscle mass assessment often prove limited, thus prompts increasing interest in ultrasound suitability.

**Methods:**

We searched the Cochrane Library, PubMed and Embase for literature published up to June 2023. Ultrasound diagnosis of sarcopenia in CKD patients was included. Two independent investigators used the Quality Assessment Tool for Diagnosis Accuracy Studies (QUADAS-2) to assess the quality. We extracted valuable information from eligible studies. Using a Bayesian bivariate model, we pooled sensitivity and specificity values and summary receiver operating characteristic (SROC) curves.

**Results:**

Five articles, involving 428 participants at various stages of CKD were included. Three studies diagnosed by the cross-sectional area (CSA) of the rectus femoris, while two others by muscle thickness (MT) and shear wave elastography (SWE) from the same muscle, separately. Overall, CSA or SWE had a pooled sensitivity of 0.95 (95% CrI, 0.80, 1.00), and the specificity was 0.73 (95% CrI, 0.55, 0.88) for diagnosing sarcopenia in CKD patients.

**Conclusions:**

Ultrasound measurements of CSA and SWE were more sensitive for diagnosing sarcopenia in the CKD population than in the general population. Ultrasound assessment from a single peripheral skeletal muscle site may serve as a rapid screening tool for identifying sarcopenic individuals within the CKD population, if a specific cut-off value could be determined.

## Introduction

Sarcopenia, the gradual loss of muscle mass and strength, causes decreased strength and limited aerobic activity [1]. It’s recognized as a systemic issue linked to conditions like diabetes, depression, cognitive impairment, and cardiovascular events. These comorbidities significantly impact quality of life and even life expectancy [2–4]. The prevalence of sarcopenia varies widely, estimated at 5–13% in individuals aged over 60 and up to 50% in those aged 80 and above [5]. These divergent findings underscore the complexity and heterogeneity of diagnosing sarcopenia. A subgroup that is particularly susceptible to sarcopenia comprises individuals with chronic kidney disease (CKD) [6]. Existing literature indicates that CKD patients have a higher risk of sarcopenia and subsequent mortality than individuals with normal kidney function [7, 8]. There is a notable decrease in muscle mass among patients undergoing hemodialysis. Chronic inflammation, metabolic acidosis and comorbidities induced by kidney failure result in the development of sarcopenia [9–11]. Therefore, early detection and prompt intervention of sarcopenia are especially needed for CKD patients.

Muscle mass assessment in the diagnosis of sarcopenia has been done using advanced imaging tools such as X-ray absorptiometry (DXA), computed tomography (CT), and bioelectrical impedance analysis (BIA) [12]. However, access to these techniques may be limited by contraindications, cost, or potential radiation exposure risk. Ultrasound has become a promising method for monitoring muscle health [13–15]. By measuring muscle thickness and utilizing innovative imaging methods like sonoelastography, which reflect muscle mechanical properties and physiology, ultrasound provides standardized and reproducible evaluations for sarcopenia diagnosis [16]. Its portability also adds the particular value for community-based assessments of sarcopenia.

The SARCUS consensuses, however, do not provide specific cut-off points for ultrasound parameters in different muscle groups to diagnose sarcopenia, primarily due to insufficient evidence [17]. There is a call for further ultrasound research to confirm its potential and clinical applicability [18]. Among CKD patients, the physical condition often varies significantly throughout different disease stages. We hypothesized that the ultrasound-based diagnosis of sarcopenia within the CKD population will display distinctions compared to other demographic groups. Hence, this meta-analysis aims to systematically review the current data concerning the diagnostic accuracy of muscle ultrasound specifically in CKD.

## Method

This meta-analysis was conducted and reported according to the Preferred Reporting Items for Systematic Reviews and Meta-Analyses (PRISMA) [19]. A prespecified protocol was registered on the PROSPERO database (CRD42023439849).

### Databases and searches

Two independent investigators conducted an electronic search of databases, including the Cochrane Library, PubMed, and Embase, from their inception through June 2023. The following keywords and the corresponding Medical Subject Headings (Mesh) terms were combined to search the databases: (‘sarcopenia’ OR ‘muscle’ OR ‘musclar’) AND (‘kidney’ OR ‘renal’ OR ‘nephropathy’ OR ‘hemodialysis’ OR ‘haemodialysis’ OR ‘peritoneal dialysis’ OR ‘MHD’ OR ‘CAPD’) AND (‘ultrasonography’ OR ‘ultrasound’ OR ‘echography’). We also screened the references of all retrieved articles for additional pertinent studies.

### Inclusion and exclusion criteria

The inclusion criteria included the following: [1] study type: prospective or retrospective observational studies involving CKD patients in whom muscle ultrasound and one of the reference standards of sarcopenia were performed; [2] study population: adult men and women of any ethnicity and in any stage of CKD; [3] index tests: any type of ultrasound that measured any muscle group in any anatomical location; and [4] outcomes that were available as true-positive (TP), false-positive (FP), false-negative (FN) and true-negative (TN).

The exclusion criteria included the following: [1] Abstracts, letters, editorials, expert opinions, reviews and case reports; [2] non-English publications; [3] articles without sufficient data for the calculation of sensitivity and specificity; and [4] duplicated publications.

### Data extraction

Two authors (CZ and ZZ) screened the titles and abstracts of the searched results independently to identify potential eligible records. Then, they reviewed the full texts of these eligible publications while following the inclusion and exclusion criteria to decide on the final studies for inclusion. If a disagreement occurred, a third independent researcher (QY) was asked to arbitrate. The reasons for exclusion were recorded. The citations used in the included studies were also screened for potentially eligible studies.

The data were extracted from the included studies by two authors independently using a structured data extraction form. The following variables were extracted: name of the first author, publication year, country, study population, sample size, age, proportion of men, diagnostic method for sarcopenia, prevalence of sarcopenia, measurement details (probe, axis, type of transducer, muscle group, detection index) and outcomes.

The Quality Assessment of Diagnostic Accuracy Studies-2 (QUADAS-2) tool was employed to assess the methodological quality of each study [20], and the report was generated subsequently in Review Manager 5.3. These procedures were also completed independently by two authors (CZ and ZZ). In the event of any discrepancy, a third investigator (QY) was brought in to resolve the dispute.

### Data synthesis and analysis

The statistical analysis was performed using a Bayesian bivariate model for diagnostic test studies. The primary advantage of this method is the ability to stabilize the analysis by incorporating a small amount of information without overpowering the existing data. This is particularly valuable when there is a limited amount of data, as the prior for the covariance matrix of the bivariate structure plays a crucial role [21]. This foundation enables the method to achieve higher accuracy [22].

The proposed method enables the direct estimation of accurate posterior marginal distributions for sensitivity, specificity, and relevant hyperparameters and covariates without the requirement of Markov chain Monte Carlo (MCMC) sampling. Additionally, it directly provides the pooled estimates of sensitivity and specificity with 95% credible intervals (CrIs) and summary receiver operating characteristic (SROC) curves, facilitating straightforward interpretation. Publication bias was assessed using the funnel plot and Egger’s statistic. All analyses were performed using R software version 4.3.1 (R Foundation for Statistical Computing, Vienna, Austria; https://www.r-project.org) and RStudio version 2023.06.0-421 (RStudio, Inc., Boston, MA, USA) with the R packages meta4diag 2.1.1 and INLA 23.06.25, along with other necessary packages.

## Results

**Fig. 1:**
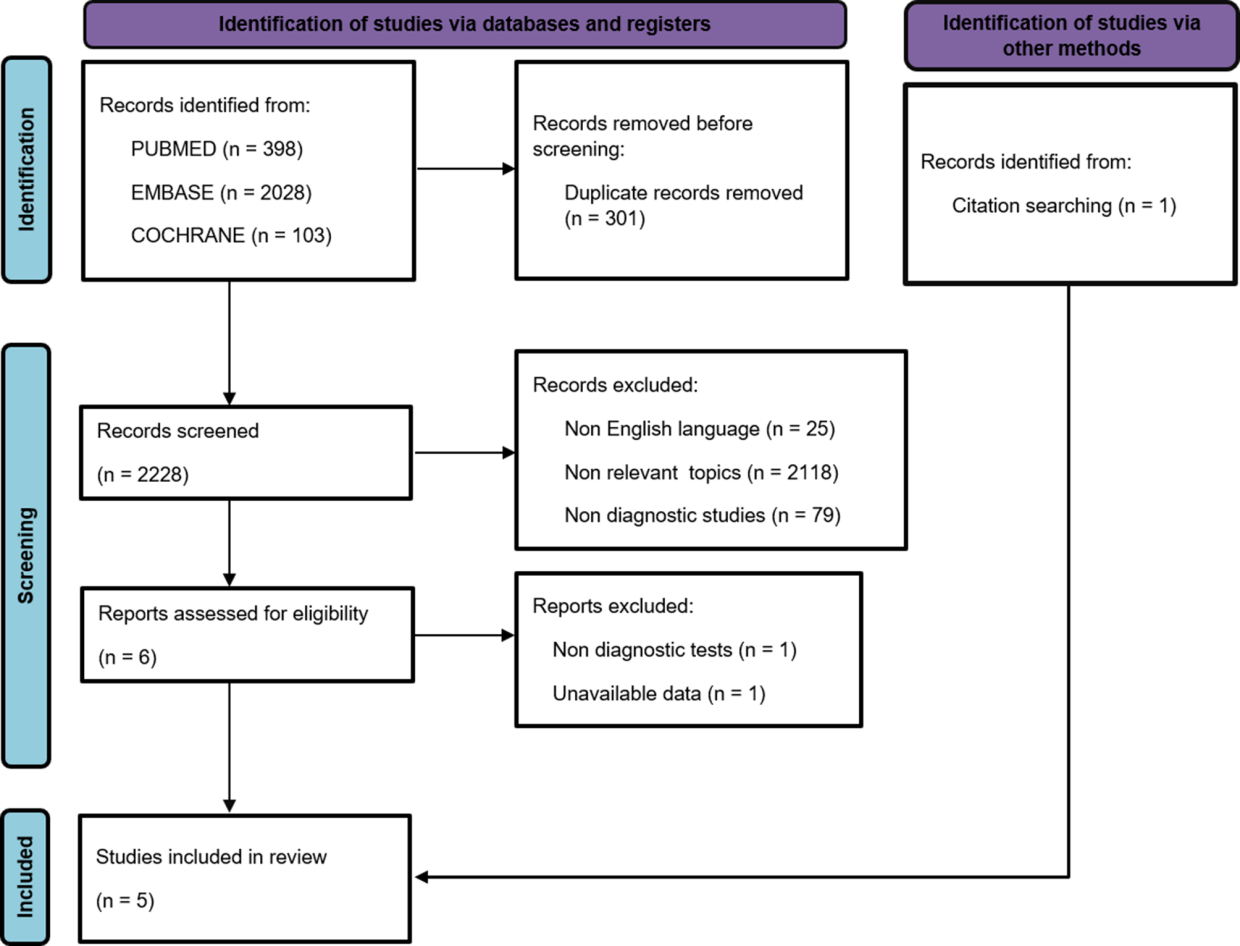
Selection of studies included in this meta-analysis

A detailed flow chart of the literature search is presented in Fig. 1. We initially identified 2529 citations from the databases and one citation from references. After removing duplicates, a total of 2228 articles remained. Screening of the titles and abstracts yielded six studies, two of which were excluded following full text review. Finally, we selected 5 articles involving 428 participants at various stages of CKD [23–27].

### Characteristics of the included studies

The main characteristics of the individual studies are summarized in Table 1, including the optimal cut-off values for diagnosis and AUC-ROC (95% CrI). They were published between 2018 and 2022 and were conducted in five different countries. One study covered patients from CKD stages 1 to 5. Two studies focused on pre-dialysis CKD patients, while another two targeted those with end-stage renal disease: one specifically investigated hemodialysis patients, and the other centered on post-kidney transplantation patients.

**Table 1:** Characteristics of studies included in the meta-analysis

Study	Country	Population	Age* (years)	N (male, %)	Reference standard	Machine model	Probe	Axis	Measured muscle	Index test	Cut-off value	AUC
Chen 2021 [27]	China	Patients who underwent kidney transplantation	17–39	40 (55.0)	skeletal muscle index by CT images	SuperSonic Imagine	Linear, 2-10 MHz	Trans, Long	RF	SWE	10.37 kPa	0.845
Souza 2018 [26]	Brazil	Patients in pre-dialysis CKD stages	73.5 ± 9.22	100 (41.0)	FNIH-defined sarcopenia	Siemens Sonoline G40	Linear, 6–12 MHz	Trans, Long	RF	CSA	M: 13.2 mm, FM:10.9 mm	M: 0.595, FM: 0.619
Wilkinson 2021 [23]	UK	Patients in CKD	62 ± 14.1	113 (47.8)	LMM-defined sarcopenia	Hitachi EUB-6500	Linear, 7.5 MHz	Trans	RF	CSA	M: 8.9cm^2^, FM: 5.7 cm^2^	M: 0.7, FM: 0.9
Matsuzawa2021 [25]	Japan	Hemodialysis patients	77.5 ± 11.8	58 (62.1)	AWGS 2019-defined sarcopenia	Toshiba Medical Xario 200	Linear, 4–11 MHz	Trans	RF	CSA	NA	NA
Rao 2022 [24]	India	Patients in pre-dialysis CKD stages	51.6 ± 14.6	117 (75.0)	AWGS 2019-defined sarcopenia	Fujifilm Sonosite Sono Site M-TURBO^®^	NA	Trans	RF	MT	M: 1.1 cm, FM:1.0 cm	M: 0.749, FM: 0.628

Abbreviations: AUC, area under the curve; Trans, transverse ultrasound scan; RF, rectus femoris; CSA, cross-sectional area; SWE, shear wave elastography; MT, muscle thickness; M, male; FM, female; AWGS, Asian Working Group for Sarcopenia, by which the diagnosis is based on muscle strength and/or low gait speed, with muscle mass measured by bioelectrical impedance analysis [29]; FNIH, Foundation for the National Institutes of Health Sarcopenia Project, by which the diagnosis is based on muscle strength and appendicular lean mass measured by dual-energy X-ray absorptiometry [28]; LMM, low muscle mass, by which the diagnosis is based on the ratio of muscle mass (measured by bioelectrical impedance analysis) to height squared or the ratio of muscle mass to BMI [33]

*Age: mean ± standard deviation

**Fig. 2:**
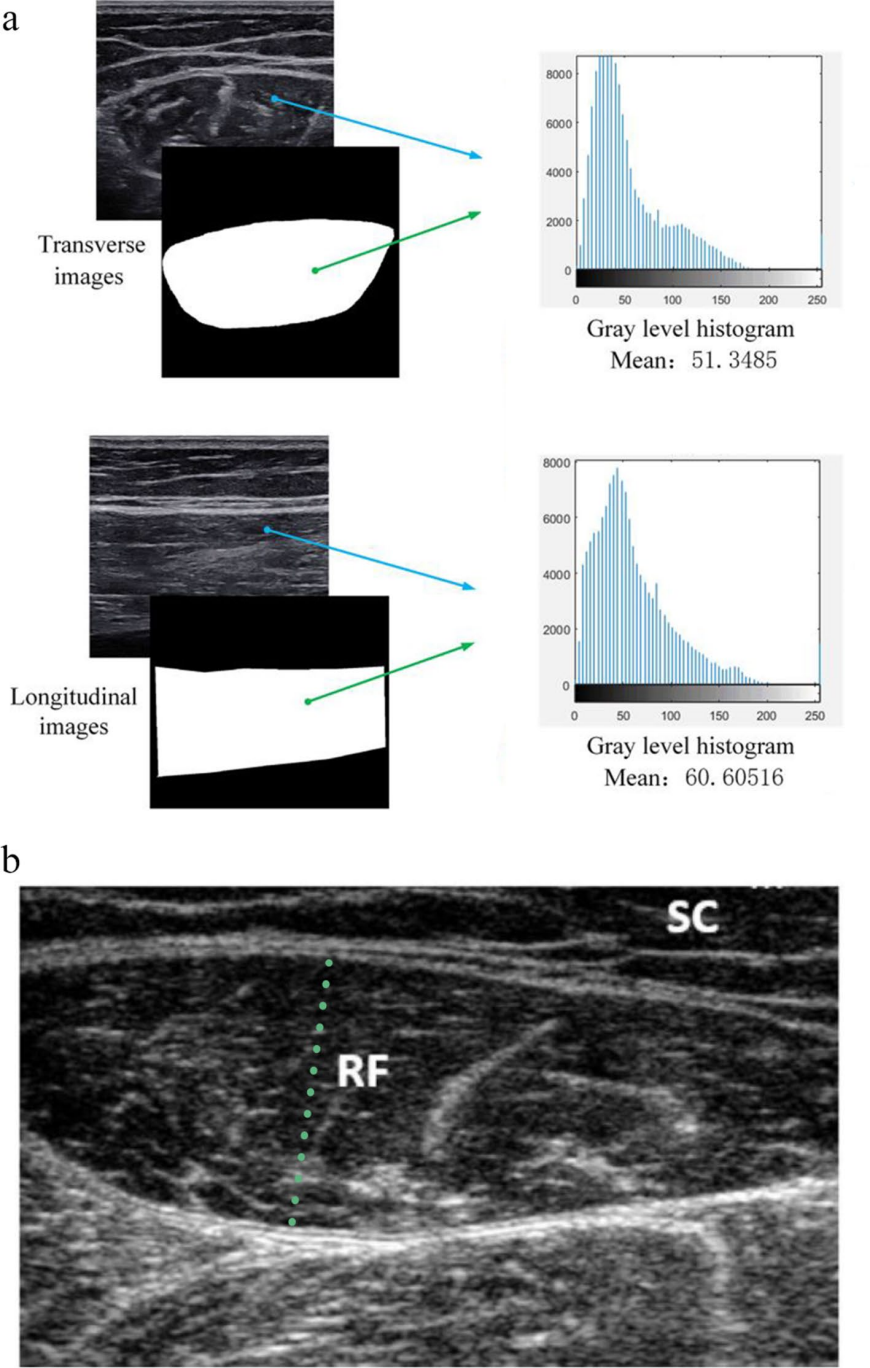
Ultrasound assessment of shear wave elastography and cross-sectional area (CSA). (a) The region of rectus femoris was selected in transverse and longitudinal images followed by the calculation with echo intensity analysis [27]; (b) CSA of the rectus femoris [35]. Copyright, *Oxford University Press* and *John Wiley and Sons*

The ultrasound measurement methods varied across studies. Among the included studies, four utilized linear array probes. The ultrasound frequencies employed ranged from 4 to 12 MHz. All included studies conducted inspections on a single site of the rectus femoris (RF), with the index tests such as cross-sectional area (CSA), muscle thickness (MT), and shear wave elastography (SWE). Figure 2 shows the ultrasound appearance under the SWE and CSA. Two studies reported the probe position in the transverse axis, while three studies reported the probe position in both the transverse and long axes. Regarding the reference standards, two studies defined sarcopenia based on the skeletal muscle index at CT images or low muscle mass separately [23, 27], and the others used the AWGS 2019 or FNIH criteria [28, 29].

### Evaluation of methodological quality

**Fig. 3:**
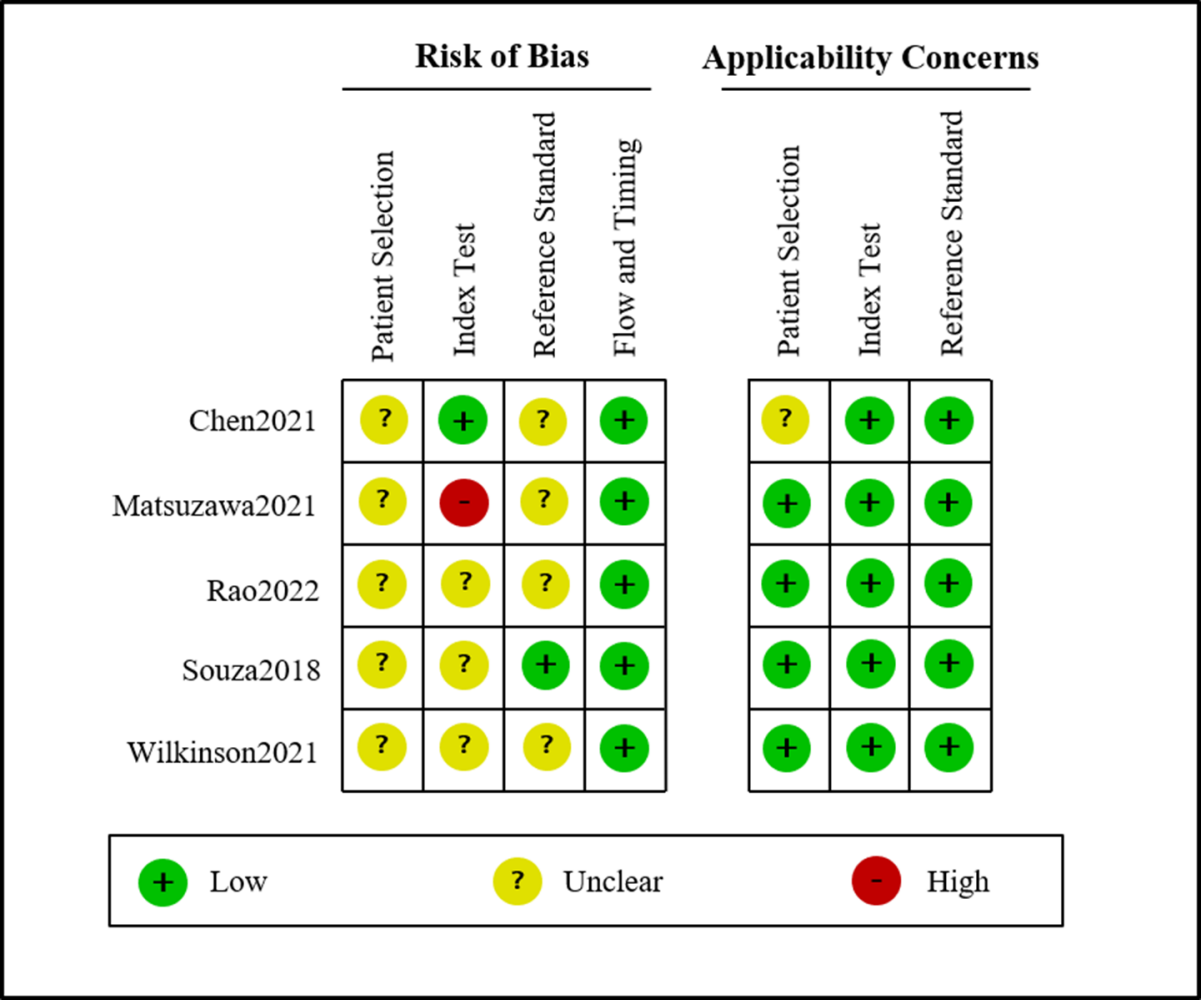
Assessment of risk of bias of studies: QUADAS-2 tool. QUADAS-2, Quality Assessment of Diagnostic Accuracy Studies-2

The results of the methodological quality assessment for the included studies are shown in Fig. 3. Most studies presented an unclear risk of bias in patient selection and reference standard domains, as they did not provide precise details about consecutive sample selection and blinded reference standard measurements. Three studies were rated as having an unclear risk of bias, and only one study had a high risk of bias in the index test domain. As for applicability concerns, one study had an unclear concern regarding the patient selection domain, while the others had low concerns across all three domains.

### Results of diagnostic test accuracy

**Fig. 4:**
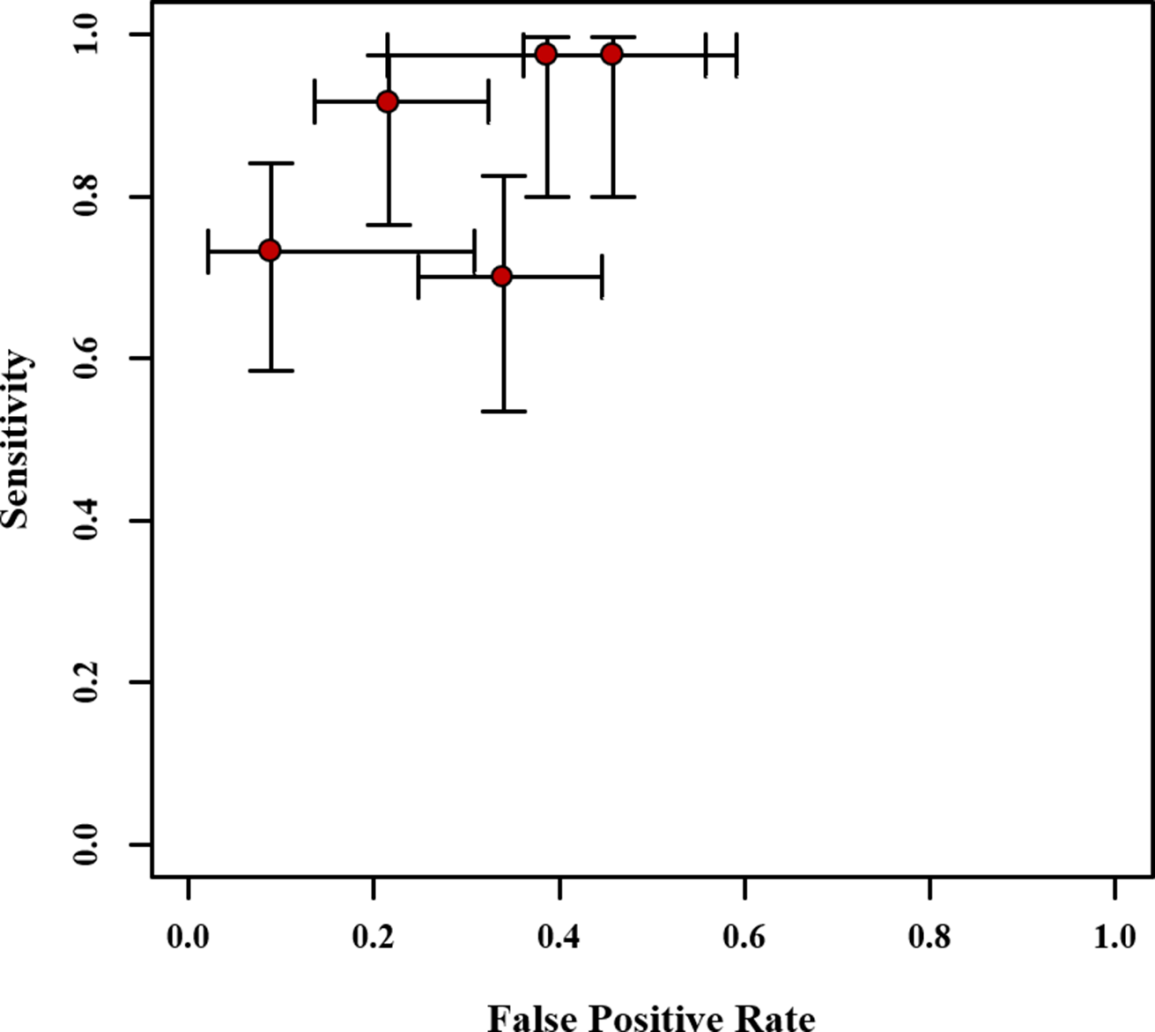
Crosshair plots of the pooled sensitivity. The estimated accuracy for each study is plotted as a red solid circle, and the 95% credible interval (CrI) is plotted as arrows

Crosshair plots showed sensitivity, false positive rate values, and confidence intervals for each included study (Fig. 4). Out of the five studies included in the analysis, one study evaluated RF-MT as a parameter for predicting sarcopenia. The bivariate analysis revealed a sensitivity of 0.73 (0.57–0.86) and specificity of 0.68 (0.58–0.77) for this particular study. These values were generally lower than the findings in the other four studies. A meta-analysis also indicated that ultrasound assessment of a single MT produced a lower diagnostic test accuracy for sarcopenia in the general population. This aligns with the results obtained in our study. Consequently, to minimize heterogeneity, the study assessing RF-MT was excluded in subsequent analyses.

**Fig. 5:**
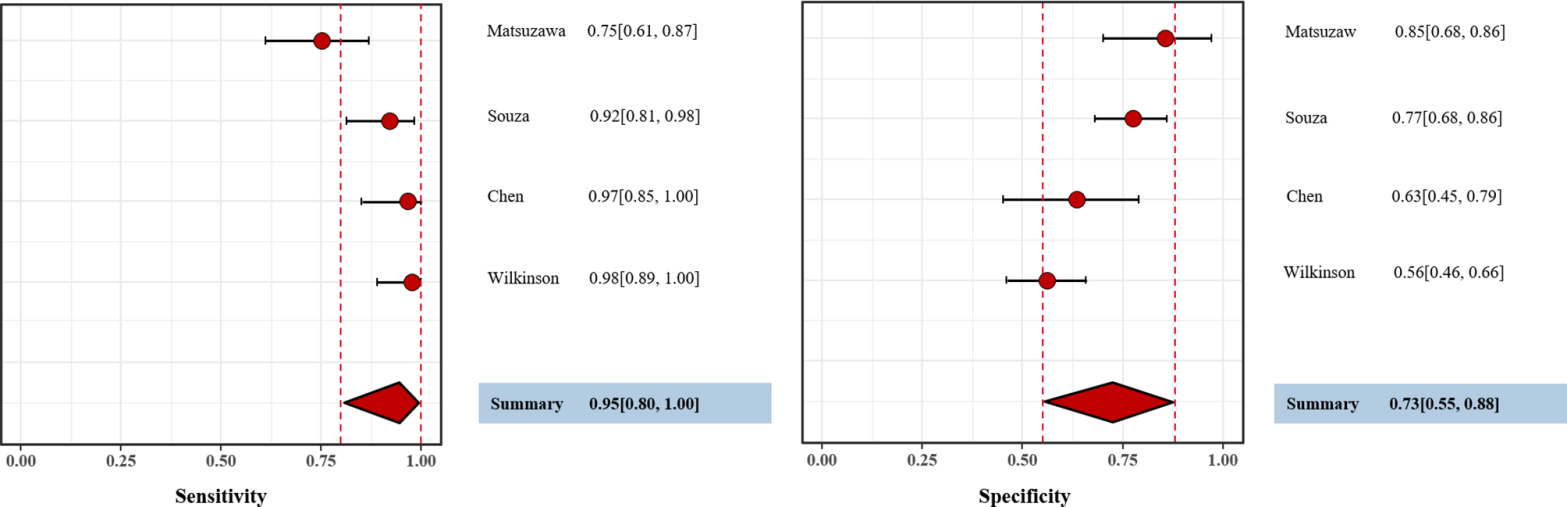
Forest plots of the pooled sensitivity (left), and specificity (right) of the ultrasound parameter in diagnosing sarcopenia. The estimated accuracy for each study is plotted as a red solid circle and the 95% credible interval (CrI) is plotted as arrows. A diamond indicates the overall summary point

**Fig. 6:**
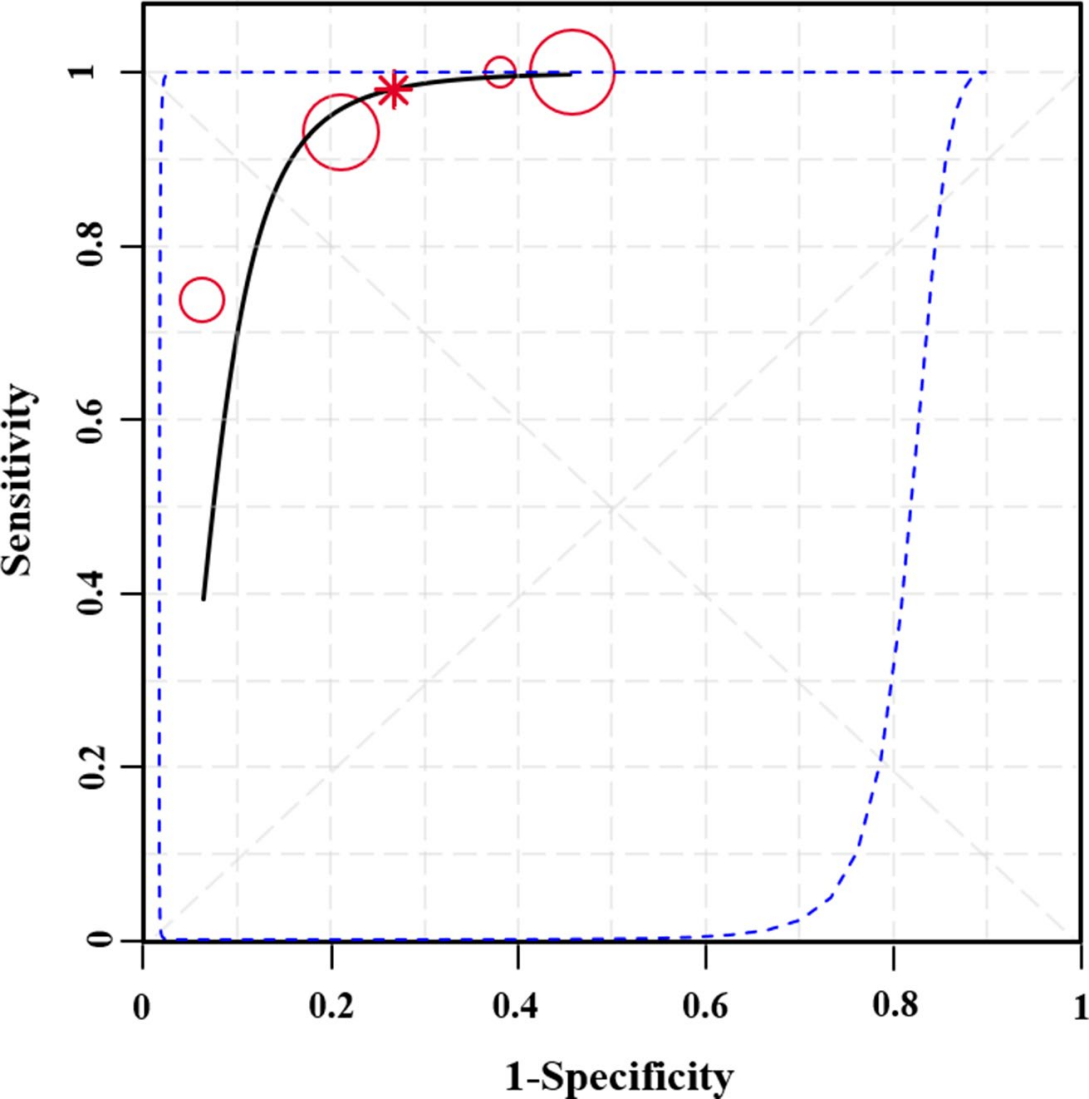
Summary receiver operating characteristic (SROC) plots of the ultrasound parameter in diagnosing sarcopenia. The summary receiver operating characteristic line is plotted as a black solid line; the summary point is marked in red; each analyzed study is represented by a circle; the area enclosed by blue dotted lines represents the confidence region

The remaining four studies were based on RF-CSA or RF-SWE, with sensitivity ranging from 0.75 to 0.98 and specificity from 0.56 to 0.85 (Fig. 5). The pooled sensitivity of muscle ultrasound in the diagnosis of sarcopenia was 0.95 (95% CrI, 0.80, 1.00), and the specificity was 0.73 (95% CrI, 0.55, 0.88). The estimated diagnostic odds ratio (DOR) was determined to be 5.15 (95% CrI, 2.41, 12.58), while the positive likelihood ratio (PLR) and negative likelihood ratio (NLR) were 3.94 (95% CrI, 1.92, 8.81) and 0.07 (95% CrI, 0.00, 0.29), respectively. The summary receiver operating characteristic (SROC) plot demonstrated that muscle ultrasound had a reasonable diagnostic efficiency for sarcopenia in CKD (Fig. 6). Furthermore, we conducted a specific analysis for RF-CSA. The results were consistent with the findings mentioned above (Table 2).

**Table 2:** Subgroup analysis for CSA studies

Study	Sensitivity (95% CrI)	Specificity (95% CrI)	+LR (95% CrI)	-LR (95% CrI)	DOR(95% CrI)
All studies (4)	0.95 (0.80, 1.00)	0.73 (0.55, 0.88)	3.94 (1.92, 8.81)	0.07 (0.00, 0.29)	5.15 (2.41, 12.58)
CSA studies (3)	0.91 (0.72, 1.00)	0.76 (0.54, 0.93)	5.24 (1.63, 17.42)	0.13 (0.00, 0.50)	4.06 (1.66, 7.89)

**Fig. 7:**
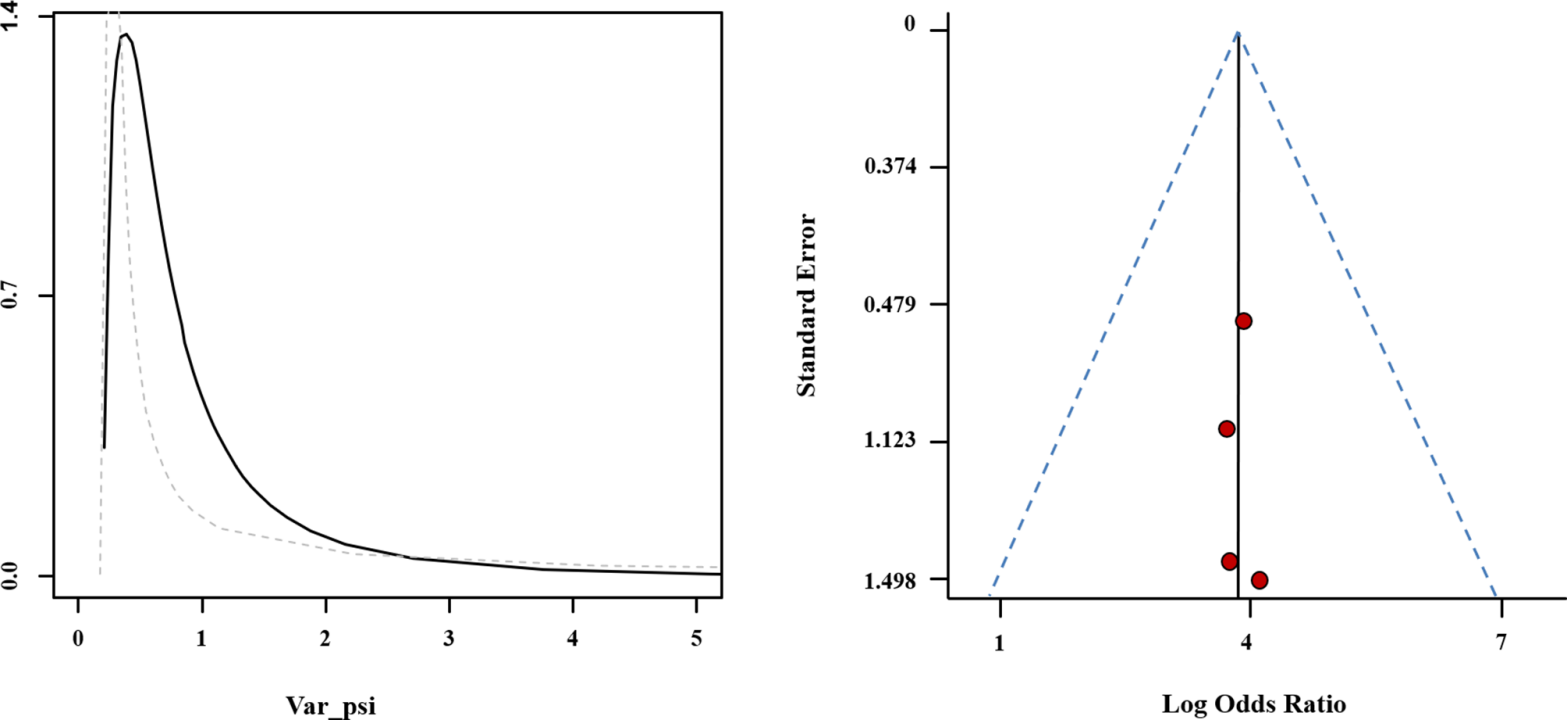
Posterior density distribution plot (left) and Funnel plot (right) for the evaluation of potential bias in the included studies. The posterior density distribution plot displays the consistency of outcomes; the closer the peak is to the coordinate [1], the more consistent the outcomes are (left). In the funnel plot the X-axis represents the diagnostic odds ratio and the Y-axis shows the index of precision of the diagnostic odds ratio (right)

Our research was based on a Bayesian bivariate model, which was stable and of good consistency, as shown in Fig. 7. No publication bias was evident for the studies of CSA or SWE. In the funnel plot, each circle representing an individual study is close to the reference line, which might be related to the small number of studies and the small sample size (Fig. 7). The Egger’s test was 0.15.

## Discussion

Sarcopenia, despite often being underrecognized in clinical practice, has become a worldwide health issue [30, 31]. As stated, many of the relevant measurement techniques for assessing skeletal muscle mass are not feasible for frail CKD patients in a community setting. These patients comprise a substantial population and require regular medical follow-ups and examinations [32]. There is a growing interest in the applicability of ultrasound. It has been proven to have good validity to estimate muscle mass compared to MRI, CT and DXA [33], although evidence on its ability to diagnose the presence of sarcopenia is limited [34]. Our study findings might help to generalize the use of ultrasound in diagnosing sarcopenia in the CKD population.

To our knowledge, this is the first meta-analysis showing the diagnostic accuracy of ultrasound-derived variables for sarcopenia in CKD patients. The CSA and SWE taken from RF showed a moderate diagnostic accuracy, whereas the MT of RF seemed to have a relatively lower accuracy. Both MT and CSA are commonly used parameters to assess muscle quantity due to their easy accessibility on the lower leg [23]. RF-CSA has been extensively studied and established to be associated with muscle function and strength in CKD [35]. On the other hand, SWE is an ultrasound parameter that reflects muscle quality. In the context of muscle ultrasound, muscle quality typically represents the relative composition of various components within muscle tissues, including muscle, blood vessels, and adipose tissue [36]. Muscle quality has been increasingly recognized as equally significant as muscle quantity in the diagnosis of sarcopenia. Researchers have noted that the values of CSA fail to take into consideration the composition of the muscle [27]. Especially in patients undergoing dialysis treatment, frailty, obesity, and fluid overload are prevalent. Our study found that the results from RF-CSA were consistent with the overall findings, suggesting similar diagnostic capabilities of RF-CSA and RF-SWE for sarcopenia in CKD.

The study’s high sensitivity indicates that ultrasound-derived variables are highly effective in correctly identifying individuals with sarcopenia. The lower specificity suggests that 27% of patients with normal muscle mass were incorrectly classified as sarcopenic. In clinical settings, physicians should be cautious when interpreting the values below the cut-point of muscle ultrasound in CKD patients, and evaluate more parameters for muscle mass with other techniques, muscle strength and physical performance. However, this still implies that point-of-care ultrasound may serve as a valuable tool for quickly ruling out sarcopenia. Given our objective of prompt and early detection of sarcopenia in CKD patients, these data support the utility of muscle ultrasound. According to the meta-analysis by Fu et al., CSA indexes demonstrated a sensitivity of 82–84% and specificity of 69–72% in diagnosing sarcopenia from the general population [37]. The sensitivity of echo intensity was 61–67%, with a specificity of 70–71%. In comparison, muscle ultrasound appears to have greater sensitivity for diagnosis in the CKD population.

To achieve higher specificity in the diagnosis of sarcopenia through ultrasound, a single parameter taken from a limb muscle site may not be sufficient. The combination of CSA and echo intensity was found to improve diagnostic test accuracy [37]. Tang et al. introduced an ultrasound scanning system that measures MTs at four muscle sites and provides an integrated estimation of the skeletal muscle mass index [38]. This comprehensive approach yielded a sensitivity and specificity of over 90% in detecting low muscle mass. Furthermore, when combined with handgrip strength and gait speed measurements, it demonstrated a diagnostic sensitivity of 92.7% and specificity of 91.0% for sarcopenia in the elderly cohort. The emphasis in sarcopenia diagnosis has shifted from pure loss of muscle mass to initial measurement of muscle strength and physical performance [33]. The future goal is to develop a composite diagnostic method for the CKD population, based on multipoint muscle ultrasound assessments, to efficiently improve the diagnostic accuracy. Additionally, the inclusion of parameters such as CSA and echo intensity in such a diagnostic system could also be considered.

Although various criteria can be used to diagnose sarcopenia and the detrimental effects of sarcopenia in CKD patients are gradually becoming evident, the methods for predicting adverse outcomes in sarcopenic patients remain uncertain. Research prioritization should focus on innovative and practical assessments that predict significant outcomes, such as mortality or end-stage kidney disease [39]. Studies have indicated that ultrasound-derived parameters on the vastus intermedius or diaphragm were independently associated with mortality or cardiovascular events in dialysis patients [40, 41]. Therefore, muscle ultrasound could potentially be used to predict clinical outcomes.

In conclusion, our study indicated that muscle ultrasound using CSA and SWE from a single peripheral muscle site exhibits better diagnostic test accuracy for sarcopenia in the CKD population, compared to previous studies conducted in the general population. Ultrasound examination may serve as a rapid screening tool for identifying sarcopenic individuals within the CKD population. However, challenges such as low specificity and the absence of defined cut-off values still need to be addressed through further methods and research.

### Strengths and limitations

This study faced limitations regarding the number and the sample size of included studies. While a stable Bayesian bivariate model was employed for diagnostic analysis, the insufficient data prevented the establishment of specific cut-off values. The included studies exhibited significant heterogeneity in ultrasound parameters, disease stages, and reference standards. Particularly concerning were the reference criteria, as some might not serve as ‘gold standard’, such as the dual-energy X-ray absorptiometry for muscle mass. Consequently, the diagnostic value of muscle ultrasound in this work is somewhat hindered by these inconsistencies. In the study by Matsuzawa [25], eight patients were unable to undergo BIA testing due to contraindications. Of these, six were diagnosed with sarcopenia by ultrasound, and all eight were considered as sarcopenia patients in our study. Data imputation could potentially affect the representation of the actual level.

## Data Availability

All data analyzed during this study are included in this published article.
